# DHA and Its Metabolites Have a Protective Role against Methylmercury-Induced Neurotoxicity in Mouse Primary Neuron and SH-SY5Y Cells

**DOI:** 10.3390/ijms22063213

**Published:** 2021-03-22

**Authors:** Ami Oguro, Kenta Fujita, Yasuhiro Ishihara, Megumi Yamamoto, Takeshi Yamazaki

**Affiliations:** 1 Program of Biomedical Science, Graduate School of Integrated Sciences for Life, Hiroshima University, Hiroshima 739-8521, Japan; m191175@hiroshima-u.ac.jp (K.F.); ishiyasu@hiroshima-u.ac.jp (Y.I.); 2 Department of Environment and Public Health, National Institute for Minamata Disease, Kumamoto 867-0008, Japan; MEGUMI_YAMAMOTO@env.go.jp; 3 Program of Life and Environmental Sciences, Graduate School of Integrated Sciences for Life, Hiroshima University, Hiroshima 739-8521, Japan; takey@hiroshima-u.ac.jp

**Keywords:** methylmercury, DHA, DHDP, RXR, ROS

## Abstract

The consumption of fish now involves a risk of methylmercury (MeHg) exposure but also provides the benefit of ω-3 polyunsaturated fatty acids (ω-3 PUFAs) such as docosahexaenoic acid (DHA). Some epidemiological studies have suggested that the intake of DHA can alleviate the neurotoxicity of MeHg, but the underlying mechanism is not known. Herein, we observed that pretreatment with 0.1–1 µM DHA suppressed MeHg-induced cytotoxicity in human neuroblastoma (SH-SY5Y) cells and mouse primary neuronal cells. These effects of DHA were canceled in the presence of the retinoid X receptor (RXR) antagonist UVI3003. An RXR agonist, bexarotene, suppressed the cytotoxicity of MeHg. DHA also suppressed the MeHg-induced production of reactive oxygen species (ROS) via an induction of antioxidant genes (*catalase* and *SOD1*). Pretreatment with DHA did not change the incorporation of MeHg. We showed previously that in the brain, the intake of DHA increased the level of 19,20-DHDP, which is the metabolite produced by cytochrome P450 and soluble epoxide hydrolase from DHA. In the present study, we observed that 19,20-DHDP also suppressed neurotoxicity from MeHg. These results indicate that DHA and its metabolites have a protective role in MeHg-induced neurotoxicity.

## 1. Introduction

Methylmercury (MeHg) is a neurotoxicant concentrated in fish and shellfish through the food chain [1,2]. In humans, MeHg exposure mostly occurs via the consumption of seafood. The formation of a complex of MeHg and cysteine allows MeHg to easily move across the blood–brain barrier and the placenta and adversely affect the central nervous system and fetal development [3]. However, the consumption of seafood also serves as an important source of beneficial nutrients including ω-3 polyunsaturated fatty acids (PUFAs) such as docosahexaenoic acid (DHA) and eicosapentaenoic acid (EPA). DHA is the most abundant ω-3 PUFA in the phospholipid content of the mammalian brain and is essential for neuronal development and optimal cognitive health [4].

A cohort study conducted in the Republic of Seychelles of the potential relationship between prenatal MeHg exposure and the maternal status of ω-3 PUFAs from fish consumption with child development demonstrated that increased prenatal MeHg exposure was associated with lower scores on the Psychomotor Developmental Index (PDI) for children only among the children of mothers with lower ω-3 PUFA value [5]. Among the children of the mothers with higher DHA value, the impairment of PDI scores due to increased MeHg exposure was lessened [5]. A cohort study of Faroese children also showed that a short delay recall in the California Verbal Test was associated with a doubling of the cord blood level of MeHg, and this association became stronger after adjustment for the values of ω-3 PUFAs [6]. These two studies suggested that ω-3 PUFAs such as DHA may attenuate MeHg-induced neurotoxicity. The molecular mechanisms underlying the mitigation of MeHg neurotoxicity by DHA remain to be clarified, however.

DHA can cross the blood–brain barrier and is accumulated in the brain as an esterified form into phospholipid. The esterified DHA contributes to the fluidity of the phospholipid membrane, whereas the free DHA produced by the activation of phospholipase A2 exerts a variety of effects by activating several receptors including the two G-protein-coupled receptors GPR40 and GPR120 [7], retinoid X receptor (RXR) [8], and peroxisome proliferator-activated receptors (PPARs) [9]. It has been shown that DHA is rapidly accumulated in the brain of the fetus after DHA supplementation during the midgestation period, but total DHA reaches a plateau and replenishes only the amount of DHA consumed in the brain from the plasma in early adulthood [10]. We have also shown that DHA supplementation in adult rats did not change the free DHA levels in the brain but increased the DHA metabolites dihydroxydocosapentaenoic acids (DHDPs) produced by cytochrome P450s (P450s) and soluble epoxide hydrolase (sEH) [11]. We also observed that DHA supplementation reduced oxidative stress in the brain, and DHDPs contributed to this effect, suggesting that DHA metabolites produced by P450s and sEH as well as DHA have important roles in this neuroprotective effect.

An increase in oxidative stress is the major mechanism that has been proposed to mediate MeHg-induced neurotoxicity [12,13]. It was demonstrated that mitochondrial reactive oxygen species (ROS) are clearly involved in oxidative stress and the subsequent cell death induced by MeHg in neuronal cells [14]. One of the mechanisms underlying the MeHg-induced generation of ROS was shown to take place through an impairment of antioxidant enzymes such as manganese superoxide dismutase (Mn-SOD) by the binding of MeHg to thiol groups of the proteins and through glutathione (GSH) depletion [12]. We thus speculated that the antioxidant effects of DHA and/or its metabolites have a protective effect against MeHg-induced neurotoxicity. We conducted the present study to investigate the effects of DHA and its metabolites on MeHg-induced cytotoxicity in a human neuroblastoma cell line (SH-SY5Y) and mouse primary neuronal cells.

## 2. Results

### 2.1. Protective Effect of DHA against MeHg-Induced Cytotoxicity in SH-SY5Y Cells and Mouse Primary Neuronal Cells

**Figure 1:**
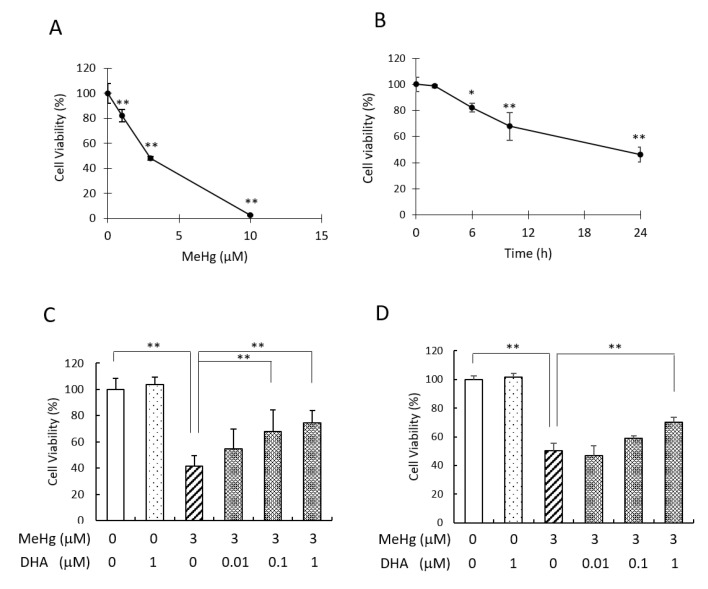
Pretreatment with DHA alleviated MeHg-induced cytotoxicity in SH-SY5Y and mouse primary neuronal cells. (**A**) SH-SY5Y cells were treated with MeHg at the concentrations of 1, 3, and 10 µM for 24 h, and cell viability was measured by MTT assay. (**B**) SH-SY5Y cells were treated with 3 µM MeHg for 2, 6, 10, and 24 h, and the cell viability was measured by MTT assay. SH-SY5Y cells (**C**) or mouse primary neuronal cells (**D**) were pretreated with DHA at the indicated concentration for 24 h, and then the cells were re-treated with DHA and 3 µM MeHg. After 24 h, the cell viability was measured by MTT assay. Values are the mean ± SD for four experiments. * *p* < 0.05; ** *p* < 0.01.

We first conducted an MTT assay to determine the dose–response of the cytotoxicity of MeHg in human neuroblastoma SH-SY5Y cells, applying the MeHg concentrations of 1–10 µM for 24 h. The viability of the cells treated with 3 µM MeHg was reduced to approximately 40% compared to the non-treated cells, and 10 µM MeHg significantly reduced the cell viability (Figure 1A). These results are consistent with previous findings in SH-SY5Y cells [14,15]. We next investigated the time–response of the cell viability by treatment with 3 µM MeHg for 0–24 h. The exposure of the cells with MeHg for up to 2 h did not affect the cell viability, and after 2 h the cell viability declined in a time-dependent manner (Figure 1B). Based on these results, we used 3 µM as the concentration of MeHg for 24 h in the subsequent experiments. To investigate the effects of DHA on the MeHg-induced cytotoxicity, we pretreated SH-SY5Y cells with DHA at concentrations of 0.01–1 µM for 24 h before MeHg treatment and then measured the cells’ viability. The results demonstrated that pretreatment with 0.1 or 1 µM DHA alleviated the cytotoxicity caused by MeHg (Figure 1C). We also investigated the protective effect of DHA against MeHg using isolated mouse primary neuronal cells. In these cells, pretreatment with 1 µM DHA attenuated the cytotoxicity of 3 µM MeHg (Figure 1D). These results suggest that DHA has a neuroprotective effect against MeHg-induced cytotoxicity.

### 2.2. DHA Treatment Alleviated MeHg-Induced Cytotoxicity via the Activation of RXR

**Figure 2:**
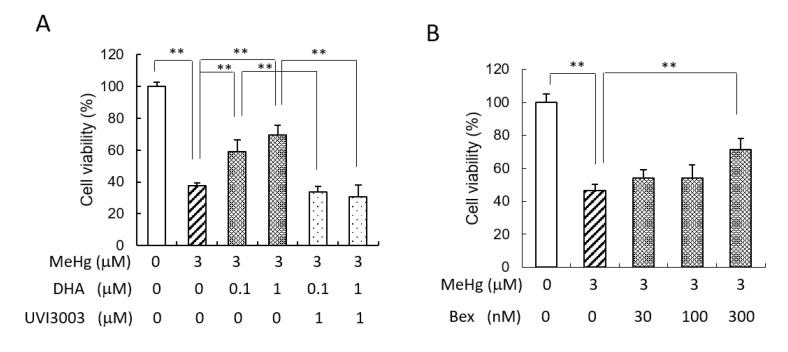
The protective effect of DHA was suppressed by an RXR antagonist. (**A**) SH-SY5Y cells were pretreated with 0.1 or 1 µM DHA along with the RXR antagonist UVI3003 for 24 h, and then the cells were re-treated with DHA and these antagonists, followed by the addition of 3 µM MeHg. After 24 h, the cell viability was measured by MTT assay. (**B**) SH-SY5Y cells were pretreated with the RXR agonist bexarotene at the indicated concentration for 24 h, and then the cells were treated with 3 µM MeHg for 24 h and the cell viability was measured by MTT assay. Values are the mean ± SD for four experiments. ** *p* < 0.01.

RXR is one of the DHA-targeting receptors, and we have observed that DHA can activate RXR in the murine brain [16]. RXRa and RXRb were shown to be expressed in SH-SY5Y cells [17]. Our present experiment showed that in the presence of the RXR antagonist UVI3003, the protective effect of DHA against MeHg-induced cytotoxicity was suppressed (Figure 2A), suggesting that DHA alleviates MeHg-induced cytotoxicity by activating RXR. Pretreatment of the cells with the RXR agonist bexarotene also showed a neuroprotective effect against MeHg (Figure 2B).

### 2.3. Pretreatment with DHA Reduced MeHg-Induced ROS

**Figure 3:**
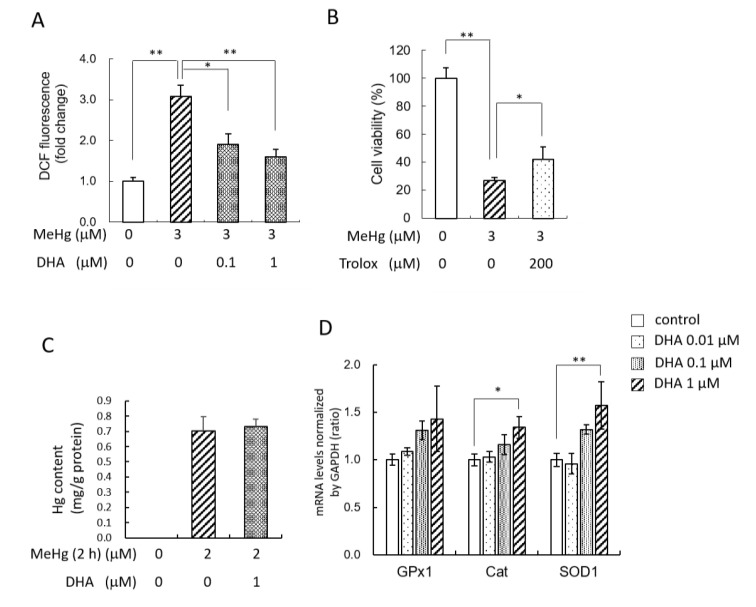
DHA suppressed MeHg-induced ROS through the induction of antioxidant genes. (**A**) SH-SY5Y cells were pretreated with 0.1 or 1 µM DHA for 24 h, and then 3 µM MeHg and DHA were added to the cells. After a 2 h incubation, the intracellular ROS levels were measured using carboxy-H_2_DCFDA. (**B**) The cells were pretreated with 200 µM Trolox for 1 h, and then the cells were treated with 3 µM MeHg for 24 h. The cell viability was measured by MTT assay. (**C**) SH-SY5Y cells were pretreated with 1 µM DHA for 24 h, and then 2 µM MeHg was applied together with DHA to the cells for 2 h. After the cells were washed with PBS, the Hg content of the cells was determined using a direct thermal decomposition mercury analyzer. (**D**) SH-SY5Y cells were treated with DHA at the indicated concentration for 24 h, and the mRNA levels of *GPx1*, *catalase*, and *SOD1* were measured by real-time PCR. Values are the mean ± SD for 3–5 experiments. * *p* < 0.05; ** *p* < 0.01.

The major mechanism that was proposed to mediate the neurotoxicity of MeHg is increased oxidative stress [18], and we have demonstrated that MeHg increased the amount of ROS derived from mitochondria in SH-SY5Y cells [14]. Therefore, we next investigated the effect of DHA on ROS levels in the present study. The intracellular ROS levels were increased at 2 h after treatment with 3 µM MeHg (at which cytotoxicity was not observed), but pretreatment with DHA at the concentration of 0.1 or 1 µM inhibited the MeHg-induced ROS (Figure 3A). We confirmed that pretreatment with Trolox (6-hydroxy-2,5,7,8-tetramethylchroman-2-carboxylic acid), a water-soluble vitamin E analog, also exerted a protective effect against MeHg-induced cytotoxicity (Figure 3B), suggesting that the suppression of ROS was one of the causes of DHA-induced neuroprotection against MeHg. We next measured Hg levels incorporated or absorbed into the cells 2 h after the addition of MeHg. Because treatment of 3 µM MeHg for 2 h slightly decreased total protein levels in the cells, we used 2 µM MeHg for the treatment. The results revealed that pretreatment with DHA did not affect the cellular Hg levels (Figure 3C). We then analyzed the mRNA levels of antioxidant genes after the addition of DHA, and we observed that 1 µM DHA significantly increased the mRNA levels of *catalase* and *SOD1* (Figure 3D), suggesting that DHA can suppress MeHg-induced ROS by inducing antioxidant genes.

### 2.4. The DHA Metabolites DHDPs Also Have Neuroprotective Effects against MeHg

**Figure 4:**
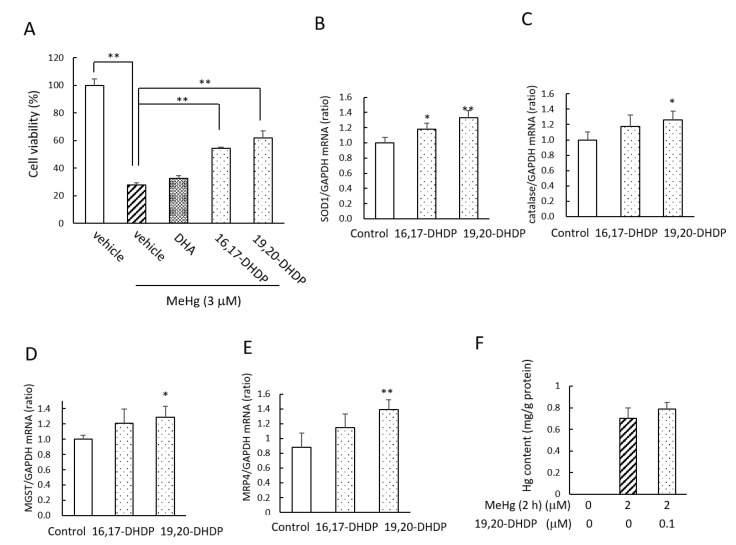
DHA metabolites DHDPs also have protective effects against MeHg-induced neurotoxicity. (**A**) Mouse primary neuronal cells were pretreated with DHA or a DHDP at the concentration of 0.1 µM for 24 h. Then, 3 µM MeHg was applied together with DHA or a DHDP to the cells for 24 h, and the cell viability was measured by MTT assay. (**B**–**E**) Mouse primary neuronal cells were treated with a DHDP at a concentration of 0.1 µM for 6 h, and the mRNA levels of *SOD1*, *catalase*, *MGST*, and *MRP4* were measured by real-time PCR. (**F**) SH-SY5Y cells were pretreated with 0.1 µM 19,20-DHDP for 24 h, and then 2 µM MeHg was applied to the cells together with 19,20-DHDP for 2 h. After the cells were washed with PBS, the Hg content of the cells was determined using a direct thermal decomposition mercury analyzer. Values are the mean ± SD for 4–5 experiments. * *p* < 0.05; ** *p* < 0.01.

In an earlier investigation using rats, we observed that supplementation with DHA increased the levels of the DHA diols (DHDPs formed by P450s and sEH) in the brain [11]. In the present study, we therefore investigated the effects of DHDPs on MeHg-induced cytotoxicity. In mouse primary neurons, pretreatment with 0.1 µM 16,17-DHDP or 19,20-DHDP significantly alleviated the decrease in the cell viability induced by MeHg, although 0.1 µM DHA did not show a protective effect (Figure 4A). We observed that 19,20-DHDP (0.1 µM) also increased the mRNA levels of *SOD1* (Figure 4B), *catalase* (Figure 4C), and microsomal glutathione *S*-transferase 1 (*MSGT*) (Figure 4D). The treatment with 19,20-DHDP induced the mRNA of multidrug resistance-associated protein 4 (MRP4) but not that of MRP1–3, which is the transporter for inorganic mercury export (Figure 4E). However, pretreatment with 19,20-DHDP did not affect the cellular Hg levels after the addition of MeHg to the cell medium (Figure 4F). These results suggest that like DHA, the DHDPs also have a neuroprotective effect against MeHg, and this effect of the DHDPs was more effective than that of DHA at the concentration of 0.1 µM.

## 3. Discussion

Our present findings revealed that DHA at the concentration 0.1–1 µM attenuated MeHg-induced neurotoxicity. Epidemiological studies in Seychelles and Faroese suggested that an intake of DHA could attenuate the neurotoxicity of MeHg [5,6]. However, in vitro experiments by Takanezawa et al. and Kaur et al. showed an enhancement of MeHg-induced toxicity by pretreatment with 5–20 µM DHA in mouse embryonic fibroblasts [19] and by 30–90 µM DHA in C6-glial and B35-neuronal cell lines [20], although the same research group also suggested the protective effect of DHA against MeHg in mouse primary neuronal cells [21]. While DHA is the most abundant in the brain as an esterified form into phospholipid, our earlier study demonstrated unesterified DHA at 0.76 nmol/g tissue of rat brain [11]. The values of 1.3 nmol/g tissue of rat brain were reported by Demar et al. [22], and 2.6 nmol/g tissue of rat brain with an adequate diet of ω-3 PUFA and 0.42 nmol/g tissue of rat brain with deficient diet of ω-3 PUFAs were reported by Igarashi et al. [23]. Therefore, the concentration of DHA used in the present study would be a physiological concentration. The DHA concentrations 0.5–1 µM were shown to have physiological functions in neuronal cells such as neurite outgrowth [24] and the induction of ApoE receptor, which reduces beta-amyloid production [25].

Our results demonstrated that the protective effect of DHA occurred via the activation of RXR. RXR is expressed in the central nervous system, and DHA was shown to be an endogenous ligand for RXR in mouse brain [26]. The activation of RXR by DHA was observed in vivo in mouse brain [27], and DHA was determined as a main constituent in the most effective fraction of mouse brain for RXR activation [28]. In addition, the EC_50_ value for RXR activation by DHA was reported to be 5–10 ìM in a study using a reporter assay with RXR-binding sites from the *apolipoprotein 1* gene and by a mass spectrometry analysis of the binding between the RXR ligand-binding domain and DHA [8]. This EC_50_ value of DHA is much higher than the physiological concentration of DHA, and thus the possibility of an indirect RXR activation by DHA was also suggested in several reports [29]. RXR can act as a homodimer or heterodimer with another nuclear receptor such as PPAR, retinoic acid receptor (RAR), nuclear receptor-related 1 (Nurr1), constitutive androstane receptor (CAR), or thyroid hormone receptor (TR) [29]. As DHA also binds to PPAR and Nurr1 [9,30], DHA may act as a dual agonist of these complexes. A strong activation of these heterodimers by ligands for either partner has also been observed [31], and thus it is possible that DHA can affect a partner of RXR in the heterodimer and determine its sensitivity or transcriptional activity. The plasma membrane receptors GPR40 and GPR120 were also identified as DHA receptors [32], but the GPR40/120 agonist GW9508 did not show a protective effect against MeHg in SH-SY5Y cells in the present study.

The production of ROS is involved in the manifestation of MeHg toxicity [12]. Our present experiments showed that DHA suppressed MeHg-induced ROS via an induction of antioxidant genes, *SOD1* and *catalase*. The in vivo experiment in our previous study revealed that supplementation with DHA in mice [16] or rats [11] induced antioxidant genes in the brain. In another of our studies, the addition of the RXR agonist bexarotene to rat hippocampal slices induced catalase and GPx1 [33]. RXR activation would thus be one of the mechanisms of the induction of antioxidant genes by DHA. We also investigated the possibility that DHA may alter the cellular accumulation of MeHg in the present study, but we observed that the Hg contents of the neuronal cells were not altered, although Jayashankar et al. observed that in mice, the maternal exposure to MeHg with DHA reduced the MeHg accumulation in the pup brain but not in the liver [34].

DHA is metabolized to bioactive mediators produced by lipoxygenase (LOX), cyclooxygenase-2 (COX-2), and P450s [35]. The LOX-mediated metabolites protectins (PDs), maresins (MaRs), and resolvins (RvDs) are known to have important roles in neuronal protection and anti-inflammatory effects [36], and DHA is also metabolized to epoxidized forms, epoxydocosapentaenoic acids (EDPs) by P450s, and further hydrolyzed to the corresponding diols (16,17-, 13,14-, 10,11-, 7,8-, and 19,20-DHDP) by sEH [37]. We have observed that 19,20-DHDP was most abundant among them in rat brain, and contributed to the neuroprotective effect of DHA supplementation in a rat model of Parkinson’s disease through the induction of antioxidant genes [11]. In the present study, a protective effect of 19,20-DHDP against MeHg was also observed in neuronal cells with the induction of antioxidant genes, and DHDPs were more effective against the cytotoxicity of MeHg than the same concentration of DHA. As our earlier study also showed that 19,20-DHDP induced the stabilization of NF-E2-related factor (Nrf2), which is involved in the induction of SOD1 and catalase in neuronal cells [11], Nrf2 may have contributed to the induction of antioxidant genes by DHDP in the present study. MeHg is incorporated in sulfur-containing cysteine to form a MeHg–cysteine complex and can be transported to cells [38,39] and exported from cells by ATP-binding cassette (ABC) transporter MRPs, which function in the efflux of glutathione conjugates of inorganic mercury. MRPs were also revealed to be Nrf2-targeting genes [40]. After myogenic cells’ exposure to MeHg with the MRP inhibitor Ceefourin 1, the Hg content was significantly higher than that in non-treated cells [41]. Our present results demonstrated that 19,20-DHDP increased the mRNA levels of MRP4, but the induction would be insufficient because no alteration of adsorbed Hg in the cells by pretreatment with 19,20-DHDP was observed.

The present study suggests that an intake of DHA together with MeHg can mitigate the neurotoxicity of MeHg, and our findings revealed for the first time that the DHA metabolites DHDPs also have a neuroprotective effect. We reported that DHDPs were produced by P450s and sEH in adult rat brain [11], and further research is necessary to clarify the effects of DHDPs on the MeHg-induced neurotoxicity in vivo. On the other hand, the expression levels of these enzymes were shown to be low in the brain of infant rodents [42,43]. Therefore, further investigations to clarify the production in fetal brains of DHA metabolites and their effect on maternal MeHg exposure will help combat the high susceptibility of fetal brains to MeHg neurotoxicity.

## 4. Materials and Methods

### 4.1. Reagents

Dulbecco’s modified Eagle’s medium (DMEM) was purchased from Sigma Chemical (St. Louis, MO, USA). Penicillin–streptomycin solution was purchased from FUJIFILM Wako Pure Chemical Corp. (Osaka, Japan). Fetal bovine serum (FBS), neurobasal medium (phenol red and glutamine minus), and B27 supplement were obtained from Thermo Fischer Scientific (Waltham, MA, USA). Bexarotene and triciribine were obtained from Cayman Chemicals (Ann Arbor, MI, USA). UVI3003 was purchased from Tocris Bioscience (Bristol, UK). Methylmercury chloride was obtained from Tokyo Chemical Industry (Tokyo, Japan). Carboxy-H_2_DCFDA was obtained from Molecular Probes (Eugene, OR, USA).

### 4.2. Isolation of Murine Cortical Primary Neuron

Mouse primary cortical neurons were prepared from ICR mice (SLC, Inc., Shizuoka, Japan) on the 16th day of gestation as described [44]. The cortex was separated, and the meninges were removed. The tissues were cut into small pieces and then dissociated using a papain dissociation system (Worthington Biochemical, Freehold, NJ, USA). The resulting cell suspension was filtered through a cell strainer (40 μm, Falcon, Tewlsbury, MA, USA) and plated on polyethylenimine-coated dishes with neurobasal medium and B27 supplement. After 2 days of culture at 37 °C in 5% CO_2_ and 95% air, cytosine β-d-arabinofuranoside (Sigma) was added to inhibit glial proliferation (final concentration, 1 μM), and the medium was changed completely after 2 days to remove the cytosine β-d-arabinofuranoside.

### 4.3. Culture of SH-SY5Y Cells

The human neuroblastoma cell line SH-SY5Y was obtained from the American Type Culture Collection (ATCC, Manassas, VA, USA) (cat. No. CRL-2266) and maintained in DMEM supplemented with 10% FBS, penicillin (100 units/mL), and streptomycin (100 μg/mL) at 37 °C in 5% CO_2_ and 95% air. For the cell treatment of DHA or DHDPs, culture medium was replaced with DMEM containing 0.5% FBS 1 day after seeding of cells.

### 4.4. Measurement of Cell Viability

Cell viability was measured by a 3-[4,5-dimethylthiazol-2-yl]-2,5-diphenyl-tetrazolium bromide (MTT) assay. MTT was added to the cell-culture medium at a final concentration of 0.5 mg/mL. After the cells were incubated at 37 °C for 1 h in a 5% CO_2_ and 95% air atmosphere, the resulting formazan was dissolved in a 40 mM HCl/isopropanol solution. The absorbance was read at 570 nm with a microplate reader (Bio-Rad Laboratories, Hercules, CA, USA). The percentage of cell survival was calculated with the value of the untreated cells taken as 100%.

### 4.5. Measurement of ROS Levels

The ROS levels inside cells were determined using carboxy-H_2_DCF-DA as a fluorescent probe, as described [14]. Cells were incubated with 10 µM carboxy-H_2_DCF-DA for 15 min. The cells were then washed twice with ice-cold PBS, and the fluorescence was measured by a Flex Station reader (Molecular Devices, Sunnyvale, CA, USA) at an excitation wavelength of 504 nm and emission wavelength of 525 nm.

### 4.6. MeHg Treatment and Determination of Total Hg

SH-SY5Y cells were pretreated with 1 µM DHA or 0.1 µM 19,20-DHDP for 24 h, and then 2 µM MeHg was applied with DHA or 19,20-DHDP to the cells for 2 h. After the cells were washed with PBS for three times and centrifuging (800× *g*, 4 °C, 5 min), the cell suspension was diluted with 0.1% l-cysteine in PBS. The total Hg concentration in the cell suspension was determined with a direct thermal decomposition mercury analyzer (MA-3000; Nippon Instruments, Tokyo, Japan) as previously described [45,46].

### 4.7. Isolation of RNA and Reverse-Transcription RNA

Total RNA was extracted from murine primary neuronal cells or SH-SY5Y cells with RNAiso Plus (TaKaRa Bio, Shiga, Japan) following the manufacturer’s instructions. RNA purity was checked using the A_260_/A_280_ ratio, and we confirmed that the values were 1.9–2.1. RNA integrity was checked with the 28S/18S ribosomal ratio by 1.5% agarose gel electrophoresis. Single-stranded cDNA was synthesized from 1 μg of total RNA according to the ReverTra Ace protocol (Toyobo, Osaka, Japan). A real-time PCR was performed using a CFX Connect instrument (Bio-Rad) with TB Green Premix Ex Taq II (TaKaRa). PCR protocol was started: denaturation step (95 °C, 1 min), cycling program (95 °C, 5 s; 55 °C, 10 s; 72 °C, 15 s), and melting curve analysis. We confirmed that contamination of genome DHA in the isolated RNA was so small by RT-PCT without reverse transcriptase. The primers for human *GPx1* were 5′-TATCGAGAATGTGGCG-3′ (forward) and 5′-TCTTGGCGTTCTCCTG-3′ (reverse); those for human *catalase* were 5′-CTCCGGAACAACAGCC-3′ (forward) and 5′-ATAGAATGCCCGCACC-3′ (reverse); those for human *SOD1* were 5′-CTGTACCAGTGCAGGT-3′ (forward) and 5′- CCAAGTCTCCAACATG-3′ (reverse); and those for human *GAPDH* were 5′-GAGTCAACGGATTTGG-3′ (forward) and 5′-TTGATTTTGGAGGGAT-3′ (reverse). The primers for mouse *SOD1* were 5′-GGAACCATCCACTTCG-3′ (forward) and 5′- CCCATGCTGGCCTTCA-3′ (reverse); those for mouse *catalase* were 5′-ATCACCAGATACTCCAAGGC-3′ (forward) and 5′-TGACTCTCCAGTGACTGTGG-3′ (reverse); those for mouse *MGST* were 5′-GGTGAAAAGTCCCAGA-3′ (forward) and 5′-TCAAATGACTGAATCC-3′ (reverse); the primers for mouse *MRP4* were 5′-TGCATACAGCTTATGGCTAC-3′ (forward) and 5′-CTGCACGTGGTAGAAGTACA-3′ (reverse), and those for mouse *GAPDH* were 5′-AAGGGCTCATGACCACAGTC-3′ (forward) and 5′-CAGGGATGATGTTCTGGGCA-3′ (reverse). We confirmed that DHA does not affect *GAPDH* mRNA levels; hence, *GAPDH* was used for normalization of PCR data.

### 4.8. Statistical Analyses

The statistical analysis for single comparisons between means was carried out with Student’s *t*-test. For multiple comparisons, we performed a one-way ANOVA followed by Holm’s post-hoc test. Statistical analyses in Figure 1A,B were carried out with one-way ANOVA followed by Dunnett’s post-hoc test. BellCurve for Excel (Social Survey Research Information Co., Ltd., Tokyo, Japan) was used for analysis. For all results, probability *p* values < 0.05 were considered significant.

## Data Availability

The data presented in this study are available on request from the corresponding author.

## Abbreviations

MeHg	methylmercury
PUFA	polyunsaturated fatty acids
DHA	docosahexaenoic acid
RXR	retinoid X receptor
ROS	reactive oxygen species
DHDP	dihydroxydocosapentaenoic acids
